# Putting reins on the brain. How the body and environment use it

**DOI:** 10.3389/fnhum.2014.00795

**Published:** 2014-10-09

**Authors:** Dobromir G. Dotov

**Affiliations:** EuroMov, Movement to Health Laboratory, Université Montpellier-1Montpellier, France

**Keywords:** cognitive neuroscience, neurodynamics, metastability, embodiment, ecological psychology, enactivism, synergetics, dynamical disease

## Abstract

Radical embodied cognitive neuroscience (RECN) will probably rely on dynamical systems theory (DST) and complex systems theory for methods and formalism. Yet, there have been plenty of non-radical neurodynamicists out there for quite some time. How much of their work fits with radical embodied cognitive science, what do they need RECN for, and what are the inconsistencies between RECN and established neurodynamics that would have to be resolved? This paper is both theoretical hypothesis and review. First, it provides a brief overview of the typical, purely structural considerations why the central nervous systems (CNS) should be treated as a nonlinear dynamical system and what this entails. The reader will learn about the circular causality enclosing brain and behavior and different attempts to formalize this circularity. Then, three different attempts at linking dynamics and theory of brain function are described in more detail and criticized. A fourth method based on ecological psychology could fix some of the issues that the others encounter. It is argued that studying self-organization of the brain without taking its ecological embedding into account is insufficient. Finally, based on existing theoretical work we propose two roles that the CNS has to be fulfilling in order to allow an animal to behave adequately in its niche. In its first role the CNS has to be enslaved easily by patterns of behavior that guide the animal through its environment. In the second role the brain has to flexibly switch among patterns, what can be called the *metastable circuit breaker*. The relevance of this idea is supported using certain motor symptoms of Parkinson’s disease (PD). These symptoms can be explained as consequent to an excessive stability of the (metastable) circuit breaker.

## Preliminaries

Embodied approaches to cognition comprise some of the most interesting and fresh developments in the cognitive sciences in the past two decades (Brooks, 1991; Varela et al., 1991; Chiel and Beer, 1997; Clark, 1997; Anderson, 2003). The field at large, however, failed to use its momentum in order to completely dispose of certain intransigent issues such as the representational nature of cognitive architectures. This led to what was seen as the need for a *radical embodied cognitive science* based on the joint theoretical and methodological footing of *dynamical systems theory* (DST) and *ecological psychology* (Chemero, 2009).

The present issue dedicated to the topic of *radical embodied cognitive neuroscience* (RECN) is positioned on a comparable trajectory. Brain is the object of scientific scrutiny receiving by far the most attention and funding^1^ nowadays, at least in the mind and behavior sciences. Most of this research is directed at investigating how the brain implements a putative formal symbol manipulation system while an embodied approach would most likely argue that the brain engages dynamically, not symbolically with a field spanning also the body and its environment (see Chemero, 2009; Barrett, 2011; Wilson and Golonka, 2013). In the nooks and crannies of the extensive neurosciences one can find research programs that would fit comfortably under the embodied/embedded/enactive/situated or dynamical designation. An inconclusive list of these programs will be presented below along with an evaluation of their deeper theoretical commitments, or lack thereof, in the context of traditional issues in cognitive science and perception.

The main goal of the present article, however, is to anticipate a particular issue that RECN will face, an issue that is of critical importance. Spelled succinctly, the problem is that RECN cannot claim uniquely to itself the mathematical theories of nonlinear dynamical systems and complexity that it will most likely use to define its modus operandi; these have been deeply rooted in the neurosciences for decades. Radical embodied cognitive neuroscience cannot be a brand new paradigm in a Kuhnian sense unless it can explain why it is the proper theoretical backdrop for DST and self-organization in the neurosciences. Notice that this is a very different objective from the one that the dynamical perspective in the cognitive sciences (van Gelder, 1998) had to accomplish. There, the goal was to argue for the replacement of computational metaphor and tools with dynamical ones. Here, dynamics has been introduced long time ago, even helped neuroscience earn a Nobel prize, and multiple prophets can claim authority. Accordingly, the objective is not so much to bring dynamics to cognitive neuroscience as to motivate a neurodynamics that is more ecological, less isolated in the head. In particular, RECN needs to negotiate with three types of neurodynamicists who benefit from a rich track record. We will designate these as: (a) psychology- and philosophy-agnostic methodological neurodynamicists; (b) computational Bayesian prediction neurodynamicists; and (c) neo-Gestaltist neurodynamicists.

The paper is organized as follows. In Section The Brain as a Dynamical System the case for dynamics and self-organization in the neurosciences is defended briefly. In Section Breeds of Neurodynamicists the reader is introduced to some of the extant traditions by way of a gallery consisting of four cases studies. This survey also serves to reinforce the point that dynamics and self-organization have not necessarily always led everyone to consider a radical theoretical stance, at least not overtly. Aguilera et al. (2013) correctly remark that with few exceptions, their studies using simulated agents being among them, neurodynamical work tends to treat the brain in isolation and in this way ignores the richer dynamics that one would expect in fully coupled sensorimotor and environment systems. More importantly, these richer dynamics are not merely more interesting but they can be shown to serve as solutions to problems the real animals face. In Section Dynamics in the Brain is Not Enough. Self-Organization Occurs at the Ecological Scale the claim is presented that dynamics and self-organization of the brain cannot even be understood without also taking an ecological perspective into account. To modify without permission a popular adage from the earlier days of the ecological movement, in order to understand what happens inside the brain one has to understand what the brain is inside (Mace, 1977). Ashby (1962) definition of self-organization proves very useful in supporting this argument more formally. Section What is the CNS for? proposes two positive ideas about the roles that the dynamical brain could be fulfilling. First, the brain has a propensity to fall into a slave mode where behavior and the environment take the lead. Second, the brain relies on metastability to flexibly switch between different slave modes.

Two clarification notes about the style and scope of the present article are necessary. The first one is about the usage of specialized concepts. This is a theoretical review and it is beyond the means of the present paper to define and explain the terminology. There will be some mathematical terminology but no actual math. It is assumed that the reader is familiar with basic concepts in DST, mathematical and physical self-organizing systems, and complexity. It is also beyond the means of the paper to decide which of the many strands in DST, self-organizing systems and complex systems is most appropriate for RECN. Anyone who has ventured into the world of complexity knows that it is a wild jungle that is growing much faster than it is being mapped. There is only one way to be linear but there are many ways to be non-linear. This complicates our task. Yet, a greedy approach is going to be assumed here and all of these strands will be lumped together and referred to collectively and equivalently either as *DST*, *dynamics*, *nonlinear sciences*, *sciences of self-organization*, or *complexity*. Where possible, the meaning of a given concept will be constrained implicitly by including a reference to a key figure. So, “complexity” means one general thing but *complex systems* (Haken, 1988) and *complex systems* (Rosen, 1991) mean slightly different things.^2^ Finally, a radically interdisciplinary approach is assumed, one that does not respect the boundaries between scientific domains. The reader should be prepared to move from neuroscience, to an applied mathematics domain, and then to old-time cognitive science and psychology (the ones that did not care so much about the brain) in consecutive sentences without being warned. Such is the reality of modern complexity sciences.

Second, in terms of range of animal capacities concerned, it would be perfectly satisfactory if the hypotheses plausibly apply to an agent perceiving its environment and acting appropriately to find food and avoid danger, but not much more. Consider the functional capacities of a lizard. The study of consciousness, purposeful behavior, language, reasoning, imagery, etc., will have to be put for later. This is important to keep in mind for some of the claims and hypotheses to be introduced below could appear very unintuitive.

An explanation of what movement has to do with the brain is paramount. Llinás (2001) argues that the control of movement is the prime evolutionary driving force in the development and perfection of the brain. To illustrate the case, he tells the story of the sea squirt that moves around in the water and once it finds a spot with favorable conditions where it can plant itself and not move for the rest of its life it eats its brain. The ecological approach has been maintaining all along that movement and action functionality is fundamental in the greater scheme of the entirety of the human capacities (Michaels and Carello, 1981; Turvey et al., 1981; Turvey and Shaw, 1995). Thankfully, mainstream neuroscientists, in the sensorimotor domain at least, are beginning to realize this as well (Cisek, 2007; Wolpert, 2011). For the purposes of the present paper movement is understood not merely as the efficient displacement of a limb from point A to point B, but as action—the real-time control of movement that is intentional^3^ with respect to a perceived affordance in the environment.

## The brain as a dynamical system

### Structural properties

The term *neurodynamics* can be used to designate the study of brain as a dynamical system (Atmanspacher and Rotter, 2008). The argument that the brain *has* to be considered first of all as a dynamical system (Kelso, 1995; van Gelder, 1998) must have been made a great number of times. What are the most obvious reasons why the brain must be dynamical? To begin with, the brain possesses all the characteristics of dynamical systems, and many of these make no sense in human-built computers as we know them nowadays. Not to bore the reader, only a simple list follows here. First, the brain is massively *distributed* and *parallel*. In this sense it resembles a high-dimensional *network*. Network is what one calls a dynamical system when it starts having many degrees of freedom. The brain is *nonlinear* in at least two different ways: the response of at least some neurons to pre-synaptic potentials is a nonlinear (*sigmoid*) function and the brain is full of *feedback loops*, instantiating a *recurrent* network. A neuron is an *analog machine* because even the slightest pre-synaptic activity changes the distance of the neuron state from the action potential threshold. Hence, the brain is a real-time continuous-state^4^ recurrent network. The spreading of activation takes time. Dynamical systems theory has *delay equations* for that. Like everything in biology, neurons too are messy; they do not run on ideal trajectories like machined mechanical devices. Whether one should call this *stochastic dynamics* or *chaos* is not so clear yet but there are dynamics for both types. To conclude, the brain is a continuous state high-dimensional recurrent nonlinear stochastic and/or chaotic dynamical system, among other things. A Turing machine is neither of these.

### Coordination dynamics

The brain is known to exhibit some patently dynamical phenomena which are treated collectively as *coordination dynamics* (Kelso, 1995). One such phenomenon, a phase-transition from anti-phase to in-phase phase-locked mode of coordination between coupled oscillators, has been observed in the cortex (Fuchs et al., 1992). Further investigation revealed a range of more subtle dynamical regimes such as multi- and meta-stability (see also Section What is the CNS for?) in addition to simple phase-locking (Kelso, 2012; Kelso et al., 2013; Tognoli and Kelso, 2014). The entrainment of spontaneously oscillating units covers a whole range of phenomena in the brain (Buzsaki, 2006).

The emergence of macro-scale temporal-spatial patterns due to micro-scale activity is a typical nonlinear dynamical phenomenon. Even more specific to self-organizing systems, in some cases it can be shown analytically that the macro-scale patterns (indexed by way of so-called order parameters) control the activity of their own constitutive micro-scale substrate (Haken, 1978, 1988). The method of explaining brain function in terms of *order parameters* is a signature of synergetics and is of central importance for RECN.

### The slaving principle: collective variables run the show

To grasp the gist of the present article, one has to understand the notions of *collective variable*, *order parameter*, and the *slaving principle*. The technical term that Haken uses for low-dimensional patterns emerging at the level of collective behavior is *order parameter*. Reduction of dimensionality is an essential marker of self-organization. The notions of order parameter and collective variable are extended from their usual application to quantities used in “simple” physical systems (i.e., a homogeneous substance). These quantities serve to define the state of the system in terms of how constrained the individual atoms are by the other atoms. For example, the given order parameter in a gas is equal to 0 because the atoms move independently from each other whereas in the solid state of the same substance the parameter is a positive number because the atoms are *ordered* by being rigidly *coordinated* with each other. What is special about an appropriately selected order parameter is that it must clearly index the change of state of the substance. When one cools the substance to turn it into liquid and then solid state it is said to go through *phase-transitions* at the critical points and at exactly those points the order parameter exhibits a discontinuous jump. To use another popular example, a ferromagnet suddenly switches from being magnetized to not magnetized along with a gradual increase of temperature and this sudden switch in the property of magnetization is accompanied by a sudden shift in the order parameter describing the alignment of the elementary atomic magnets called spins. In the former case the spins are aligned, in the latter case they point in random directions. The arrangement happens spontaneously and globally through a sort of escalating positive feedback that passes via the macroscopic variable—the order parameter.

Part of the program of Haken’s synergetics is to show rigorously that exactly the same mechanism explains a range of phenomena at any scale of nature, including the brain, where multiple interconnected units of behavior assembled into an open, nonequilibrium system enter into a cooperative collective mode of action. Arguably, the spontaneous synchronization among neurons in a section of neural tissue obeys the same abstract principles. This has been shown in some cases where the theory and analytical methods of the physics of open, nonequilibrium systems were applied rigorously and a detailed quantitative account of the empirical phenomenon at the meso-scale (phase-transition in the cortical wave dynamics recorded during a coordination dynamics task involving an oscillatory acoustic stimulus and oscillatory motor behavior) was obtained based on the properties of the micro-scale substrate and the acoustic stimulus (Jirsa and Haken, 1996).

Strogatz (2003), another prominent proponent of such universal principles for spontaneous synchronization, surveyed the development of the Kuramoto model. It is one of the most well-studied mathematical formalisms for collective cooperative behavior mediated by synchronization. There, an array of coupled oscillators spontaneously synchronizes and the effect is shown to be mediated by a variable defined at the collective level (Strogatz, 2000). This formalism has been applied to cortical waves (Frank et al., 2000).

To get a better appreciation of how an order parameter might exercise top-down influence on the elements constituting the collective, consider the following revealing example offered by Hofstadter (2007). Think about driving a car on an open highway. You have several degrees of freedom: speed, lane, stopping on the side, etc. As the traffic gets denser, you start to be more constrained but still you are the master of your journey within the constraints. You will arrive at different times depending on how aggressively you drive. Beyond a certain critical density of cars, however, a traffic jam becomes inevitable. Then all cars start and stop as the traffic jam permits them. You creep forward in discrete jumps and how fast you accelerate within these brief periods does not affect the overall speed with which you will traverse the jammed area. You have lost your degrees of freedom to the traffic pattern. Yet, the traffic pattern is but the movement of the cars on the road!

## Breeds of neurodynamicists

A comprehensive review of all branches of neurodynamics would require a series of papers. For this reason, this part of the paper is limited only to identifying three important *types* of research programs classified on the basis of how they treat certain classical issues in perception. Furthermore, each class is introduced by way of a case study mostly focusing on an important paper or person. A fourth program, one that typically would not be recognized as neurodynamical, suggests a solution to some of the problems that the other three encounter.

### Case study 1: The psychology- and philosophy-agnostic neurodynamicists

A report in the journal *Science* by Kaschube et al. (2010) introduced an ambitious empirical and modeling study on the morphology of orientation columns within the visual cortex of three species. The particular combination of species is interesting because they evolved the same topology independently; the sameness is not due to shared genetic lineage. The main finding is that only certain abstract principles of self-organization (embodied in a particular order parameter field model), not genetic lineage nor environmental pressure during development (because even dark-reared animals developed the same structure), could account for the convergence in topology. The authors’ job is commendable for achieving not only qualitative (*similar* pattern of arrangement of orientation preference in the real and simulated tissue) but also quantitative agreement. Quantitative instead of merely qualitative agreement in the practice of dynamical modeling of biological or behavioral processes is a difficult task that can rarely be fully accomplished. In short, the study embodies several positive features that a dynamically oriented RECN aspires for.

To put the reader in context, the model that was used was a version of a Swift-Hohenberg equation, a spatial pattern formation system. It evolves in time and two spatial dimensions and makes spirals, hexagons, stripes, pinwheels, etc., sometimes called Turing patterns. It has been used to study chaos in the Rayleigh-Bénard convection instability (Cross et al., 1994) and associative memory as pattern completion (Frank, 2012). It belongs to a larger family (Hutt, 2007) including the reaction-diffusion equations. These were pioneered by Turing (1952) and can model many other real-world systems such as, for example, chemical oscillators (Haken, 1978) and neural fields (Hutt, 2007). This family of systems is a good representative of what many a dynamicist dream for: an explanation in an abstract form that applies in an universal fashion to phenomena in many different domains of science.

Unfortunately, after having taken advantage of the power of self-organization to explain a natural phenomenon at the intersection of biology and perception, Kaschube et al. (2010) fail to recognize a problem in treating the tissue as symbolic in its interaction with the rest of the brain. Self-organization at the developmental scale explains the local topology of the columns but formal symbol manipulation explains how they function within the greater context of perception: they represent to the rest of the brain a particular feature of the external world (p. 1114). This instance of “piecewise” self-organization is remarkable and disappointing at the same time. Notice that the particular pattern of arrangement is not even essential for perception; not all mammals have the same range of patterns (i.e., pinwheels) of arrangement of the columns. On the other hand, the really important questions such as what drives the functional organization of these columns, how features of different modalities are integrated given that they are treated by separate dumb and functionally specialized modules, i.e., how the rest of the brain gets to know that column A means 45° and column B means −45°, how the rest of the brain knows that this 45° is to be applied to figure C and this −45° to figure D are not deemed a worthy issue. It is as if you own a space ship but only use it to freight small packages around town. Accordingly, an important task for an aspiring RECN is to show that it is not a trivial thing to superimpose dynamical and symbolic frameworks and to steer the attention of fellow neurodynamicists to the really important questions. Parallel to that, a crucial task for the established neurodynamicist would be to show that a the mixing up of dynamics and symbolic computation does not necessarily lead to a contradiction.

With his model of pattern formation on the coat of certain animals (chemical morphogenesis) Turing (1952) pioneered a whole new brand of computation, different from the computation performed by the Turing machine. Cooper (2012a) calls it *embodied computation*^5^ and argues that Turing did not venture into it out of mere curiosity but understood the difficulties with Turing computation. Unfortunately, he did not have time to complete his more recent quest. Also, he did not have time to explain what the reaction-diffusion formalism has to do with the functional role of the skin patterns (to serve as disguise from prey or predators) and with the theory of natural selection. The study on orientation column ordering seems to follow the same trajectory.

### Case study 2: Bayesian predictive neurodynamics

In a large review paper Clark (2013) introduces us to a yet another new wave of cognitive science. It is the science of the Bayesian predictive brain. Some other aspects of Clark’s vision are intriguing. Imagine the human as fundamentally temporal all the way down to the design principles of her perceptual system; her existence is defined in terms of her future, a future made of action possibilities. This sounds like it could have come from any enactivist (Varela et al., 1991), phenomenology-inspired (Kelly, 2002; Rietveld, 2008), or in some cases even Gibsonian (Turvey, 1992; Stoffregen, 2000) ontology of perception. Maybe Clark got the ontology right?

Clark also brings a Karl Friston angle to the framework and this is what makes it dynamical and interesting within the goals of the current Topic. Friston (2010; Friston et al., 2010) enriches the Bayesian statistical framework with self-organization driven by a concretely defined potential function. The dimension of the potential function is *free energy* but it is really just the prediction error quantified not by way of variance but by way of entropy.^6^ The predictive brain, then, is a machine that likes to build models of the sensory input and probabilistically evaluates it while the error drives the movement. The ultimate goal of the model is that it is in resonance with the upcoming sensory stimulation at any point in time.

Trying to take Von Helmholtz’ ideas–cognition as hypothesis making and testing–and squeeze them in perception^7^ is bothersome. Leaving aside that small problem of abduction (Fodor, 2001) that no one knows how to solve, the tricky part is that predicting the future movement in terms of hypothesis testing means that the agent’s brain spends most of its time dealing not with action possibilities but with action impossibilities (bad hypotheses)! Lots of them have to be filtered out first. In realistic circumstances the set of options is always open, and then each possibility might lead to a dead end one step further. To be functional in a real world one has to be sensitive to context (Rietveld, 2012a,b). Context-sensitivity is non-locality but computation is local. Maybe in restricted domains the Bayesian predictive brain can brute-force its way from predicting perceptions to predicting movements and actions and vice versa. But it is not so clear whether the framework can scale up from simple laboratory tasks to the real world. Finally, Clark’s paper does not make it very clear how predictions over movement and predictions over perception in the brain are to be integrated, which is essential if the two are to work together.

Yet, we should not make a big fuss about the predictive Bayesian brain. The reason why is that maybe a lot about the project is correct and some troublesome details can be reinterpreted. Before we explain how, first consider what an ecological theory of being directed at the impending future could look like (Stepp and Turvey, 2010). Described very cursory, *strong anticipation* (Dubois, 2001, 2003) deals with the ability of chaotic dynamical systems to synchronize with other systems, to do so in an anticipatory manner when the coupling has a delay, and to maintain the synchronized trajectory some time after the coupling is removed. In very few words, statistical prediction is replaced with dynamical anticipation.

Social scientists like to look for differences between theories; mathematicians like to look for similarities. What is similar between the predictive Bayesian brain and strong anticipation? First, on a purely semantic level, prediction and anticipation mean the same thing. They might point to diverging implementations, but how diverging? Entropy-based measures of de-synchronization can be used for coupled dynamical systems^8^ (Tass et al., 1998; Pikovsky et al., 2003) and entropy-based measures are used for prediction error (Friston, 2010). This means that the entropy in the Friston model could be just a measure of asynchrony or lack of entrainment. Accordingly, a relevant hypothesis is that minimizing free energy in Friston’s formalism is the same as minimizing phase mismatch between coupled systems in strong synchronization.

### Case study 3: NeoGestalt neurodynamicists of nonequilibrium thermodynamics

Ask a Gestalt psychologist the following question, Why does one perceive X? The response would be, Because the neural tissue organized itself into an X following a gradient towards equilibrium in the tissue. Gestalt theory of perception was a theory of the brain as a system self-organizing after the principles of equilibrium thermodynamics (Stadler and Kruse, 1990). Back then they could imagine how self-organization is at play in the cortex. They could not imagine how the same principles could apply simultaneously across brain, body, and environment. Interestingly, the brain-body-environment is the field in which Merleau-Ponty wanted to embed the perceiver in order to fix Gestalt psychology. Dreyfus (1996, 2007) calls it a *body-environment gestalt*. For example, while one is playing tennis, “a gestalt is made up from the court, one’s running opponent, and the oncoming ball” (Dreyfus, 1996).

Grounding the Gestalt project in the brain failed but, one would say, maybe the failure was not due to bad theory but to bad analytical methods; there was no nonlinear dynamics at the time and no nonequilibrium thermodynamics (Stadler and Kruse, 1990). Modern theories of nonequilibrium systems such as synergetics make it possible to give Gestalt neurodynamics another try. “The order-parameter are ultimately the thoughts” (Haken, 1978, p. 15) where what is meant by order-parameter is strictly based on neural activation. This is the point where Haken and his followers made a mistake.

By moving up the imaginary scale from brain and behavior to “mind” one crosses into a completely different realm. The distinction (between macro-scale “mind” and meso-scale “brain”) is not merely of scale but also of metaphysical dimension. It is not the same sort of distinction as the one between laser substrate and laser beam (coherent lasing mode, one of Haken’s proper examples of order parameters enslaving a substrate). A philosopher would protest against the cavalier way in which a old and difficult problem is being solved in just one sentence by attaching the word “mind” to the pattern of a neural field. In addition to being poorly motivated, the idea of treating mental content as the upper-most and ultimate macro-scale pattern faces the risk of slipping into a solipsist theory of perception and consciousness. A careful read of Haken and his followers reveals that to the extent that they can explain phenomena such as multistable perception and “formation of meaning”, they rarely make use of anything outside of the brain to do so (Kruse and Stadler, 1995).

### Case study 4: Ecological extension of synergetics

An ecological interpretation can save Haken’s approach. It would do so by properly finding the place of the presumed supra-brain order-parameter dynamics not in an abstract substance of mind but at the level of the agent-environment (physical) system. The latter is of the same metaphysical kind as brain and body, just larger. Over the years, the so-called neo-Gibsonians have been developing a collection of primitives for theories of perception, action, and motor control. These primitives are selected to be congruent with the physics of the real world at the animal scale, what they called ecological physics (Gibson, 1960), and also with the physics of nonequilibrium systems (Kugler et al., 1980; Shaw et al., 1982; Kugler and Turvey, 1987). Although such an ecological re-interpretation with an eye on brain dynamics has never been attempted, the framework has been applied fruitfully at the behavioral level (Warren, 1984; Warren and Wang, 1987; Carello et al., 1992; van der Kamp et al., 1998; van Rooij et al., 2002; Richardson et al., 2007; Frank et al., 2009, 2010; Lopresti-Goodman et al., 2011, 2013; Dotov, 2013). To do so, one takes a strictly top-down approach assuming that a given pattern of perception and action performed by a participant in a given experimental setting is described by an order parameter and the order parameter dynamics along with phase transitions and other nonlinear phenomena are modeled mathematically.

A good synthesis of the ecological and synergetic theory is provided by Warren (2006). In his schematic, the dynamics of perception and action consist of a closed loop that couples agent dynamics and environment dynamics. In the one direction the coupling consist of a mapping from body movement to forces acting on the environment. In the other direction, the coupling consists of an optic (or else) array specifying the state of environment *relative* to the agent. All of this makes a dynamic field embedding multiple parts (eyes, musculoskeletal system, nervous tissue, ground, surfaces, light reflected from those surfaces, optic flow, and all the rest). The emergence of an order parameter (a behavioral dynamic pattern), i.e., a certain type of movement of the agent across the environment, means that the parts as a collective have entered a collaborative mode. This mode is indexed by a given order parameter. The behavior enslaves the parts and the parts support the behavior. With respect to a special role, if any, that the brain could be playing in this schema, see Section What is the CNS for? below.

If one can make sense of this reconceptualization of the way the parts of the body serve behavior, one will discover that it leads to a range of unintuitive realizations. First, an agent does not think the behavior, prepare the pattern, and then perform it. Instead, the behavior grabs the agent because the suitable conditions happened to occur (Shaw et al., 1982). A necessary member of these conditions is that a match exists between abilities of the agent and affordances of the environment, also known as *duality of constraint between animal and environment* (Shaw and Turvey, 1981). Second, the sequence of involvement of the parts does not necessarily have to be fixed for a given behavioral pattern to be initiated. For instance, a given pattern does not have to always start from the environment-to-agent coupling, or from within the agent’s brain. In some cases an agent might even find itself on the phase space trajectory of a given pattern completely accidentally. I might realize that I can get my cup of coffee sitting on the other side of the room and then initiate a walking behavior to displace myself towards the coffee or I might find myself walking in the appropriate direction for some other reason and then realize that I can get some coffee. From which direction in phase space one approaches a given limit set is not important as long as one gets close enough to the attractive domain. Similarly, some parts will be critical for a given pattern whereas others are replaceable. The behavior, being a nonlinear dynamic pattern, possesses certain resistance to perturbation.

### In conclusion

Is there a systematic way of distinguishing among the four flavors of neurodynamicists mentioned so far given that they all talk about the same thing—dynamical systems? One can ask the questions, What is *the* system? and, What are *the* parameters? The flavors then can be classified based on the answers. The four types of design, following the case studies in respective order are as such.

- The brain is the system. It also stores its own parameters. It runs a hybrid of continuous dynamics enclosed in boxes that are themselves embedded in sequential formal symbol manipulation.
- The brain is the system. It also stores its own parameters, adjusting them to better match a guess about the external world. In addition to being dynamical, and symbolic, it is also an abduction machine.
- The brain is the system. It is a fully self-organizing system and is self-contained. The environment provides a control parameter that is defined independently from the animal and in this sense is linearly separable.
- The system is the dynamical field spanning the brain, body, and environment. The brain, being the most flexible and adaptive part, serves to interrupt a given field or complete different fields spanning subsets of the full range of possible configurations of the environment and the body.

## Dynamics in the brain is not enough. Self-organization occurs at the ecological scale

If one is convinced that the dynamical approach is anti-reductionist, one would be disappointed to find out that nonlinear dynamics in itself does not preclude reductionist thinking. The idea behind reductionism is that higher-order properties can be obtained deductively from lower-order properties. Is not the Human Brain Project (Markram et al., 2011) the ultimate reductionist Laplacian dream? The simulations specify cellular parameters (molecular ones will come in the next years), runs a sequence of deductive operations on a Turing machine (a very long sequence indeed), and hopes to understand human behavior, perception, thinking, etc. In the core of these operations one finds the equations of motion approximating in computer discrete time nonlinear dynamical models such as reaction-diffusion equations, the Hodgkin–Huxley model (see Section Case Study 1: The psychology- and Philosophy-Agnostic Neurodynamicists for a discussion of the significance of such models).

A radical claim would be that we actually already know most of what we need to know about the brain, there is no hidden neural code to be decoded. Among the successes of the Blue Brain Project (precursor to the Human Brain Project and a big endeavor in its own right) after painstakingly detailed dynamical modeling of neuronal cortical columns one finds emergent properties at the collective level of the full column (Markram et al., 2011) such as propagating waves of synchronous activation. Interestingly, the same can be achieved using a relatively small system of dynamical equations running on your laptop, not on a supercomputer (Izhikevich, 2007). The point is not to disparage the achievements of the project. On the contrary, if propagating waves and such were among the main results of the project, they would serve to confirm an important hypothesis. The hypothesis is that neural tissue in itself does not exhibit much more than spontaneous synchronization and other phenomena typical of nonlinear dynamical systems.^9^ Augmenting the size of the simulation and taking larger and larger chunks of tissue is not going to change that, contrary to von Neumann’s (1966) hypothesis that a critical number of parts exists past which an automaton becomes self-complicating, the “threshold of complexity” idea. The interesting behavior appears when the tissue is embedded into what it has been evolved to be embedded into—a field of environmental and bodily activity—implying that the proper unit of analysis includes brain, body, and its niche.

To develop further the case of embedding the brain through the body into a niche, consider Ashby’s (1962, originally published 1962) notion of self-organization. His argument appears similar to the one developed here. First, he makes a distinction between organization in a purely structural sense and the normative quality attributed to the organization. The former is the state *S* defined by the parts of the system arranged in a certain coordinated pattern and possessing certain resilience to perturbation (nowadays we would call that arrangement an attractor); the latter cannot be evaluated without reference to the embedding environment. As per the (self-)organization of *S*, Ashby points out that networks can rewire themselves spontaneously and grow in structure but this notion does not address the normative side. For a system to be truly self-organizing in the normative sense, it would have to be able to redefine the way it responds to certain conditions when necessary. To use Ashby’s honestly cybernetic example of an instance of self-organization in structure and function, a child who is a fire-seeking system changes to a fire-avoiding system once having experienced the burn of the flame.

Ashby’s demonstrated that self-organization of a system (i.e., a brain) without considering its environment does not make sense. This is a strong argument against the sort of neurodynamics that only consider dynamics in the head as the source of behavior. Consider his thoughts further. If *f* (the behavior) is the mapping from state (of the brain) *S* to a new state *S*, the purpose of learning is to change the mapping *f*. For example, let *s_i_* be a state (of the brain) when the agent is located near the fire, *s*_j_—a state when touching the fire, and *s_k_*—a state away from the fire. If *f* mapped *S* from *s_i_* to *s_j_* before learning but *s_i_* to *s_k_* after learning, then conceptualizing learning as self-organization implies that *f* is also a function of *S*. For Ashby, however, this simply cannot occur for formal reasons; you cannot even write it in mathematical form. For clarity, imagine that *f* can exist in two different forms, *f_a_* and *f_b_*. Each of these functions maps *S* onto *S* in some particular way. For self-organization to occur, *S* has to determine which of the *f_a_* or *f_b_* is being used. This could only be made sense of formally if another system *α* having *f_a_* and *f_b_* as its states is included and *S* and *α* are taken to be mutually causing. Thus, Ashby (p. 117) concludes, “the appearance of being “self-organizing” can be given only by the machine *S* being coupled to another machine …” and “the part *S* can be ‘self-organizing’ within the whole *S*+*α*”.

Ashby puts quotation marks around “self-organizing”. He advises against the use of the expression because it leads to confusion. He probably felt that sacrificing the proper delineation of “system” and “environment” is too high a price to pay for a logically consistent understanding of self-organization. From an ecological perspective, however, agent-environment systems are a proper unit of analysis and blurring the boundary between the two can even be fruitful (Dotov et al., 2010). A sequence of theories of the (formal) mutuality of agent and environment, or environment property and agent property can be traced over the years (Shaw et al., 1982; Turvey, 1992; Chemero and Turvey, 2007, 2008).

Where Ashby stopped is where theoretical biologist Robert Rosen began (Rosen, 1991), trying to tackle the apparent self-causing nature of living systems. While Rosen’s understanding of complexity is far less popular nowadays than the different breeds of computational, dynamical, and statistical complexity, he might actually be on the right track. He was one of the few who grabbed the bull by the horns and directly addressed the *functional* complexity of living systems (*good self-organization*, in Ashby’s terms). In contrast, notions such as Shannon entropy, dimension, relative phase, coherence, scaling exponent, and a plethora of other mathematical quantities typically used in the characterization of complex systems all refer to structure or the evolution of structure in time, at best, but not to function. Only Haken’s (1988, Jirsa and Haken, 1996) notion of circular causality (the *slaving principle*) comes closer to Rosen’s ideas. For a modern application of Rosen’s theory to perceptual systems, see Chemero and Turvey (2007, 2008). It is very likely that something like Rosen’s discussion of *closure to efficient cause*, similar in spirit to the enactivist *autopoiesis* (Maturana and Varela, 1980), could shed light on how behavior in an environment shapes the brain which in its turn shapes behavior. Nowadays, Ashby’s sort of *S*+*α* self-organization is being applied for the purposes of testing theory by way of simulated agents and for further exploring the capacities of artificial systems (Buckley et al., 2008; Santos et al., 2012; Aguilera et al., 2013; Buhrmann et al., 2013).

## What is the CNS for?

### Brain as the slave to the animal-environment system

The idea that the brain is a physical substrate the microscopic elements of which are being enslaved by a higher-order pattern was introduced in Section The Brain as a Dynamical System. In Section Breeds of Neurodynamicists it was claimed that the slaving can come at play down from the level of behavior in an environment. In this sense, the brain, as the thing that sits between most sense organs and the muscles, has to be enslaved by a higher-order dynamic pattern for motor behavior to occur and be sustained. As a further illustration, consider the *blue collar brain* hypothesis (Van Orden et al., 2012). It rests on the suggestion that since the time scales of body and environment are slower than the time scales of the brain (but see, Turvey and Fonseca, 2014), the latter must be controlled, in the sense of be constrained, by the former.

This line of thinking is consistent with Fuchs (2011) understanding of the brain as a (very large) set of latent open loops. In given circumstances one of the loops will be closed, and thus selected out of the larger set, by a given configuration of the body and environment. He calls these open loops *dispositions*. Accordingly, the main job of the brain, at least in the context of on-line control of movement, tracking danger and prey, etc., is merely to close the circuit of a given dynamical field spanning a configuration of brain, agent, and environment. The important point is that selection of a given loop, which minimally corresponds to a specific mapping from sense organs to muscles, has to happen and the responsibility for this selection is offloaded to the animal-environment system.

### The metastable circuit breaker

The claim in the previous section was that an essential aspect of the functionality of the brain is to allow itself to be enslaved by a dynamic field spanning the animal and its surroundings. Evidently, in a changing environment this would lead to trouble sooner or later. To avoid becoming locked in a potentially unfavorable mode of behavior, the brain has to be sensitive simultaneously to multiple threats and opportunities in the environment and switch among them. But what sort of property is that? It seems to be a paradoxical requirement in terms of DST. Merely decreasing stability is not satisfactory. In order for the agent to possess behavioral stability the order parameter needs to sit on an attractor. In order to quickly switch, it needs to sit on a repeller, or change attractors frequently. Such a hypothetical regime where the system seems to occupy multiple points in phase-space simultaneously has been labeled *metastability*.

There have been several different proposed formalisms that are to embody the desired metastable property of the brain. One that conveys the idea well is cast in terms of a parametric configuration that keeps the order parameter dynamics a little past the loss of an attractor (Kelso and Engstrøm, 2006; Kelso and Tognoli, 2009). Alternatively, controllable chaotic dynamics (Hübler and Lüscher, 1989) could implement the desired characteristics more literally (Freeman and Barrie, 1994). Other proposals rely on more complicated systems that use a mechanism of self-suppression or mutual suppression or topology without attractors. This type of dynamic has been called *winner-less competition* (Milton, 2000; Rabinovich et al., 2010) or *transient* or *metastable* dynamic (Friston, 1997, 2000).

## Clinical relevance. Loss of the metastability property in Parkinson’s disease (PD)

What would cognitive neuroscience be without discussion of diseases? Maybe RECN can provide an original explanation of at least one of symptom of PD. The relevant facts are as follows. The disease is associated with the progressive loss of dopamine producing cells in the *substantia nigra* of the basal ganglia. Various motor symptoms advance early in the disease and involve a reduced ability to initiate movements and to stop them once they have been initiated (*akinesia*), stiffness of the muscles, slowness of movements (*bradykinesia*), rigid ineffective postural control, uncontrollable spontaneous movements (*hyperkinesia*), tremor, and freezing (Grabli et al., 2012). Akinesia earlier in the progression of the disease leads to difficulty in initiating a movement or a locomotory mode while the form of the movement itself might be unaffected. This implies that the patients might be capable of satisfactory gait and posture maintenance but switching among these locomotory modes, i.e., from walking to standing and from standing to walking, is problematic.

Akinesia is not an issue of preferential selectivity for one type of behavior over another because the symptoms can be symmetric–switching is hard either way.

This symptomatic description suggests that maybe the disease can be considered as a *dynamical disease* (Glass and Mackey, 1988; Milton, 2000) in that it is based on an alteration of a dynamical regime, in this case the metastable switching among behavioral patterns. In particular, the two behaviors seen as attractive regions in phase space are accessible by the patient but destabilizing the one in order to switch to the other is problematic. Interestingly, cuing helps patients initiate movements and in this way allows patients to prolong their independence. Cuing can consist of auditory or visual signals that provide structure in the environment that is easy to coordinate with, such as repetitive lines on the ground, the light of a laser pointer that one has to follow, or repetitive auditory stimuli (Velik et al., 2012; Young et al., 2014). In other words, the so-called cuing techniques provide structure that affords synchronization and coordination. Accordingly, the patients use this repetitive structure to prompt themselves to initiate movement (Grabli et al., 2012).

The “broken metastable circuit breaker” interpretation of the particular PD symptoms is consistent with the supposed role of the basal ganglia, the place where PD strikes. The healthy basal ganglia is involved in selecting and inhibiting different potential actions (Mink, 1996; Cisek, 2007; Cisek and Kalaska, 2010). The claim that this selection process is dynamical in nature is supported more specifically by a recent line of work showing that motor symptoms of PD are associated with *exaggerated synchronization* within the basal ganglia (Brown, 2007; Chen et al., 2007; Tass et al., 2012; Brittain et al., 2013). Excessive synchronization means excessive dynamical stability and is the opposite of metastability (Kelso, 2012; Tognoli and Kelso, 2014).

## Conclusion

An important objective of RECN is to motivate a *neurodynamics* that is more *ecological*, less isolated in the head (Section Preliminaries). The initial steps offered here involve Ashby’s logical argument of what *self-organization* might mean (Section Dynamics in the Brain is not Enough. Self-Organization Occurs at the Ecological Scale) and some untoward consequences from failing to realize the importance of the *environment* (Section Breeds of Neurodynamicists). How a dynamic field spanning both the animal and its environment (an *animal-environment gestalt*) could lead to functional behavior on the side of the animal was suggested in Sections The Slaving Principle: Collective Variables Run the Show, Case Study 4: Ecological Extension of Synergetics, and What is the CNS for? The *slaving principle* and the notion of *order parameter*, backed by the analytical tools and theory of synergetics, serve to give more rigorous footing to the idea that macroscopic order at the level of behavior can dictate the mesoscopic and microscopic activity of brain regions and neurons. An example of clinically relevant dysfunctional enslavement (loss of flexibility and context-sensitivity) of the brain was given in Section What is the CNS for?

The present article achieves its goals mostly by reviewing already existent theories and fitting them together. In fact, it might be fully consistent with Kelso’s long-term project of extending the relevance of coordination dynamics (see Section Coordination Dynamics) and synergetics (see Section The Slaving Principle: Collective Variables Run the Show) to brain function (Tognoli et al., 2007; Kelso, 2012; Tognoli and Kelso, 2014). The only important difference is to be found in the degree to which the hypothesis presented in the current article pursues the implication that the top-most order parameter is a behavioral pattern in the animal-environment system, not a neural field. Contrary to some of Kelso’s work (Kelso et al., 2013), an argument was present on the previous pages (see Section Case Study 3: NeoGestalt Neurodynamicists of Nonequilibrium Thermodynamics and Dynamics in the Brain is not Enough. Self-Organization Occurs at the Ecological Scale) in favor of the former.

Some 20 years ago, in defining the notion of the *organism-environment system*, Järvilehto (1998) suggested that a number of problems faced by modern psychology and neuroscience could be due to the very assumption of two separate systems. He pointed out that the two-systems assumption in psychology was largely due to pre-scientific common-sense intuition: our experience with the world seems to suggest a very strong dualism between inside the head and outside the head. He proposed that all functional behavior should be regarded as internal reorganization of the elements of the unitary organism-environment system. The specific role of neurons is not to process the stimulus but to have been organized already in such a way that the stimulus would complete a given behavioral pattern leading to desirable results (compare to *dispositions* and *open loops* in Section Brain as the Slave to the Animal-Environment System).

Jordan (2008) *wild-agents* continue in the vein of organism-environment systems in that they always already embody their own developmental context. Furthermore, his *wild systems theory* provides a detailed list of characteristics that organisms must possess in order to be capable of independent agency. Among them, the strongly nested architecture consisting of quasi-independent self-sustaining units at each level is important because it offers an avenue for thinking about the fact that in most organisms behavior happens as reorganization on a large range of scales in parallel, from neuron to animal-environment system.

The current work has implications for at least one standing question. The *extended mind* and *extended cognition* hypotheses maintain that cognition is massively distributed beyond the body (McClamrock, 1995; Wilson, 1995; Clark, 1997). Cognitive functions encompass not only multiple and flexibly connected brain regions (see the work inspired by the *neural reuse* and *massive redeployment* hypotheses, Anderson, 2007, 2010) but also body parts and slices of the environment (Anderson et al., 2012). A difficult question follows, however. How do all these various parts come together to work in unity for the purpose of solving a given problem? Very different configurations will be required for different tasks or for the same task performed under different conditions.

Part of the answer invokes notions such as *soft-assembly*, *interaction-dominant dynamics*, and *task-specific synergies* (Anderson et al., 2012; see also the notion of *coalitions*, Shaw and Turvey, 1981). The problem is that while these demonstrate *how* such spontaneous self-assembly of distributed cognitive devices is possible and make some very rough predictions about the statistical properties of measurements taken from these devices (Van Orden et al., 2003), they do little to explain exactly what drives the self-assembly. Spontaneous order is not sufficient for functional organization. “Good” self-organization occurs in the agent-environment system, as Ashby (1962) already pointed out half a century ago (see Section Dynamics in the Brain is not Enough. Self-Organization Occurs at the Ecological Scale). On the other hand, the schematic offered in the present article (see Sections The Slaving Principle: Collective Variables Run the Show, Case Study 4: Ecological Extension of Synergetics, and What is the CNS for?), that of an order parameter defined over a dynamic field spanning the agent and its surroundings and enslaving the components, could be pointing to a solution to this problem.

An interesting question arises when one considers a situation where the role of environment for an agent is played by another agent. It is beyond the means of the present article, however, to discuss the growing field of social coordination dynamics. It is sufficient to point that studies seeking to determine the neural dimensions of emergent agent-agent systems are beginning to appear (see, Iizuka and Ikegami, 2003; Tognoli et al., 2007; Dumas et al., 2010).

## Conflict of interest statement

The author declares that the research was conducted in the absence of any commercial or financial relationships that could be construed as a potential conflict of interest.

## Footnotes

^1^An American Decade of the Brain was proclaimed in 1990 by the then head of state and last year another Decade of the Brain was proclaimed. In Europe, a notable example of the gold rush for scientific facts that brain research represents is the industrial-scale Human Brain Project (Markram et al., 2011) set to benefit from funding so ample that it requires scientific notation to express.

^2^Both approaches are discussed further down the pages. As an example of how the two might differ in an important way, Haken’s conception of complexity tends to be more structural whereas Rosen’s is a more functional. The former focuses on the circular causal connections between parts and wholes, between microscopic and macroscopic levels of description of systems, be it physical or biological. The latter focuses on the closed causal relations among the functions of an organism.

^3^Motor comportment has its own intentionality (Kelly, 2002).

^4^If it is analog then it can also be regarded as digital if it needs be, but the other way around does not work.

^5^Cooper (2012a,b,c) explains that the original formulation of computation is not adequate when applied to the real world; work on the theory of computation did not complete in the 1940’s. Supposedly, Turing would have agreed to that; part of the motivation behind his work on chemical morphogenesis was to create a biologically-relevant theory of computation. Anyone who believes that the brain does Turing computation or that emergent patterns are Turing-machine phenomena should think again.

^6^Entropy is another measure of variability, but with a fancy sounding name. Given that Gaussian assumptions are made in a few places in the model, could it be that variance would have worked just as well as an entropy-based measure?

^7^Making Helmholtz sound Gibsonian must be a fun activity!

^8^Take two independent uncoupled oscillators freely running at different frequencies. The relative phase between them goes everywhere and as you sample it eventually it will fill a flat distribution spanning the whole range. Such a distribution will give you the most entropy you can get for the given amount of sampling. On the contrary, any sort of synchronization, even if it is transient, will produce some inhomogeneity in the relative phase distribution which will reduce its entropy.

^9^Of course the brain performs special work by, for instance, transforming the optical array dimensionally so that the muscles can accept it Pellionisz (1986). But this is different from the sequential information processing usually assumed. Here we are only going to discuss the global property of connecting and breaking the behavioral field.
